# Hybrid Modification Strategies of Metal–Organic Frameworks: A Review on Structural Design and Environmental Applications

**DOI:** 10.1002/open.70197

**Published:** 2026-04-30

**Authors:** Samar H. Elagamy, Adel M. Michael, Reem H. Obaydo, Hayam M. Lotfy

**Affiliations:** ^1^ Department of Pharmaceutical Analytical Chemistry Faculty of Pharmacy Tanta University Tanta Egypt; ^2^ Charles Sturt University “Casual Academic” Wagga Wagga Australia; ^3^ Chemistry Department Faculty of Pharmacy Ahram Canadian University Cairo Egypt; ^4^ Analytical and Food Chemistry Department Faculty of Pharmacy Ebla Private University Idlib Syria; ^5^ Pharmaceutical Analytical Chemistry Department Faculty of Pharmacy Cairo University Cairo Egypt

**Keywords:** characterization, environmental, hybridization, metal organic frame MOFs, synthesis

## Abstract

Metal–organic frameworks (MOFs) have emerged as a versatile class of crystalline porous materials. They are characterized by their high surface area, tunable pore size, and diverse chemical functionalities. These features enable their application across a wide range of fields, including catalysis, sensing, separation, and environmental remediation. To further enhance their functionality, MOFs are increasingly being integrated with other materials or molecular components to form hybrid frameworks, which combine the intrinsic advantages of MOFs with complementary properties of the secondary components. This review provides a comprehensive overview of synthesis, characterization, hybrid modification strategies of MOFs and their structural design. Furthermore, the review highlights the environmental applications of MOF‐based hybrids, emphasizing their roles in pollutant removal and chemical sensing. Finally, potential future directions are explored, particularly the integration of artificial intelligence and machine learning tools to accelerate the design, synthesis, and performance optimization of next‐generation MOF hybrids for sustainable environmental applications. In addition, future research should focus on the development of green and sustainable synthesis routes, especially the utilization of biowaste and renewable resources as eco‐friendly precursors for MOF fabrication.

## Graphical abstract

This review examines how hybrid modification strategies enhance the structural design and environmental performance of metal–organic frameworks (MOFs). It outlines key synthesis routes, functionalization approaches, and integration with complementary materials, highlighting advances in pollutant removal, sensing, and sustainable remediation. Emerging opportunities involving green synthesis and artificial intelligence‐driven design are emphasized to guide next‐generation MOF development.

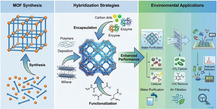

## 1 Introduction

Metal–organic frameworks (MOFs) also known as porous coordination polymers are a prominent class of crystalline materials self‐assembled through the coordination of polydentate organic ligands with metal ions or metal clusters. Their modular design allows for exceptional structural and chemical tunability, as both the metals and organic linkers can be systematically modified to achieve desired functionalities [1]. MOFs exhibit remarkable physicochemical properties, including high surface areas, adjustable pore sizes, and diverse topologies, making them attractive for a wide range of applications such as sensing, gas absorption, catalysis, and biomedical applications [2, 3, 4].

The concept of MOFs originated in 1965 when they were first observed as byproducts of chemical processes, but significant interest emerged in the late 1990s with the discovery of the first permanently porous MOFs, which subsequently led to the formal introduction of the term “metal–organic framework.” Pioneering work by Omar M. Yaghi and his team in 1995 and 1998 led to the synthesis of MOF‐5, composed of tetrahedral [Zn_4_O]^6+^ clusters linked by 1,4‐benzenedicarboxylate (BDC) ligands, forming a 3D cubic network with high porosity and surface area [5, 6, 7]. In recognition of his remarkable contributions to reticular chemistry and the establishment of MOFs as a new class of porous materials, Omar M. Yaghi was awarded the 2025 Nobel Prize in Chemistry.

The discovery of MOFs marked the beginning of a new era in porous material research, paving the way for the development of related frameworks such as IRMOFs, ZIFs, FMOFs, and MOPs, along with university‐named MOFs including UiO‐66, HKUST, and MIL series. Table 1 provides a summary of selected MOFs, their chemical compositions, and corresponding nomenclature. Around 100,000 MOFs have already been recorded in the Cambridge Structural Database [8, 9].

**TABLE 1:** Summary of selected MOFs with their compositions and nomenclature.

Name	Chemical formula/Composition	Ligand/Component	Full name/Origin
ZIF‐8	Zn(MIM)_2_	MIM = 2‐methylimidazolate	Zeolite imidazolate framework
UiO‐66	Zr_6_O_6_(BDC)_6_	BDC = benzene dicarboxylate	Universitetet i Oslo
MIL‐53	Al(OH)(BDC)	BDC = benzene dicarboxylate	Materials of Institut Lavoisier
MOF‐74	Zn_2_( DHTP)	DHTP = 2,5‐dihydroxyterephthalate	Metal organic frame
HKUST‐1 (MOF‐199)	Cu_3_(BTC)_2_	BTC = benzene‐1,3,5‐tricarboxylate	Hong Kong University of Science and Technology
IRMOF‐1 (MOF‐5)	Zn_4_O(BDC)_3_·7DEF·3H_2_O	DEF = N,N‐diethylformamide	Iso‐Reticular MOFs
F‐MOF‐1	[Cu(HFBBA)(phen)_2_](H_2_HFBBA)_2_(H_2_O)(HCO_2_)	HFBBA = hexafluorobenzeneboronic acid; phen = 1,10‐phenanthroline	Fluorinated MOF
MOP‐1	Cu_24_(m‐BDC)_24_(DMF)_14_(H_2_O)_10_	DMF = N,N‐dimethylformamide	Metal–organic polyhedra

Compared with conventional porous materials such as zeolites, MOFs possess far greater structural diversity and design flexibility. Zeolites are composed of tetrahedral SiO_4_ or AlO_4_ units, with a limited number of topological variations governed by rigid inorganic frameworks. In contrast, MOFs are built from metal ions or clusters connected by organic linkers, enabling the formation of an extensive range of 1D, 2D, or 3D architectures. This combination of organic and inorganic components imparts MOFs with larger pore volumes (often exceeding 50% of total volume), variable pore diameters (14–98 Å), low framework densities (∼0.124 g cm^−3^), and exceptionally high specific surface areas (1000–10,000 m^2^ g^−1^) [10, 11]. These attributes allow MOFs to outperform zeolites in applications requiring the diffusion or adsorption of bulky molecules. However, zeolites still maintain superior thermal and hydrolytic stability, which continues to make them indispensable in high‐temperature catalytic processes [12, 13].

MOFs also face challenges related to cost and scalability. Certain frameworks rely on expensive linkers or noble metals, limiting their large‐scale production. Furthermore, intrinsic limitations in porosity, conductivity, and steric hindrance may restrict their performance in practical applications.

To overcome these challenges, recent research has focused on hybrid modification strategies, where existing MOFs are combined with external materials or molecular components to form hybrid frameworks. These hybrid materials integrate the benefits of the parent MOF with complementary properties of other components, resulting in enhanced stability, conductivity, and catalytic or sensing performance. Covalent organic frameworks (COFs), and hydrogen‐bonded organic frameworks represent related families of porous materials that share structural similarities with MOFs and have further expanded the framework design.

The novelty of this review lies in its comprehensive coverage of the diverse hybridization strategies employed in MOF‐based materials, including their integration with polymers, nanoparticles, carbon‐based materials, and other porous frameworks. In addition to summarizing structural fundamentals and design principles, the review systematically highlights recent advances in MOF‐based hybrid systems for environmental analysis. Particular attention is given to their applications in pollutant detection, heavy metal adsorption, and catalytic degradation of hazardous compounds. Furthermore, the review critically discusses current challenges, limitations, and future perspectives associated with the development and practical implementation of MOF‐based hybrid materials in environmental applications.

### 1.1 Methods of Synthesis of Metal–Organic Frameworks

The synthesis of MOFs has been the subject of extensive research due to their diverse structural architectures and wide‐ranging applications. Achieving MOFs with high crystallinity, uniform particle size, and controlled morphology requires precise regulation of nucleation and crystal growth processes. In general, MOFs are synthesized through wet chemical routes, where the reaction involves metal ions or clusters (secondary building units) and organic linkers under specific thermodynamic and kinetic conditions. The formation process follows the classical LaMer mechanism, encompassing precursor dissolution, supersaturation and nucleation, followed by steady crystal growth until equilibrium is achieved. Reaction parameters such as temperature, solvent polarity, concentration, pH, and the presence of modulators or surfactants play crucial roles in determining the final morphology, particle size, and crystallinity of the frameworks [14, 15, 16].

**FIGURE 1:**
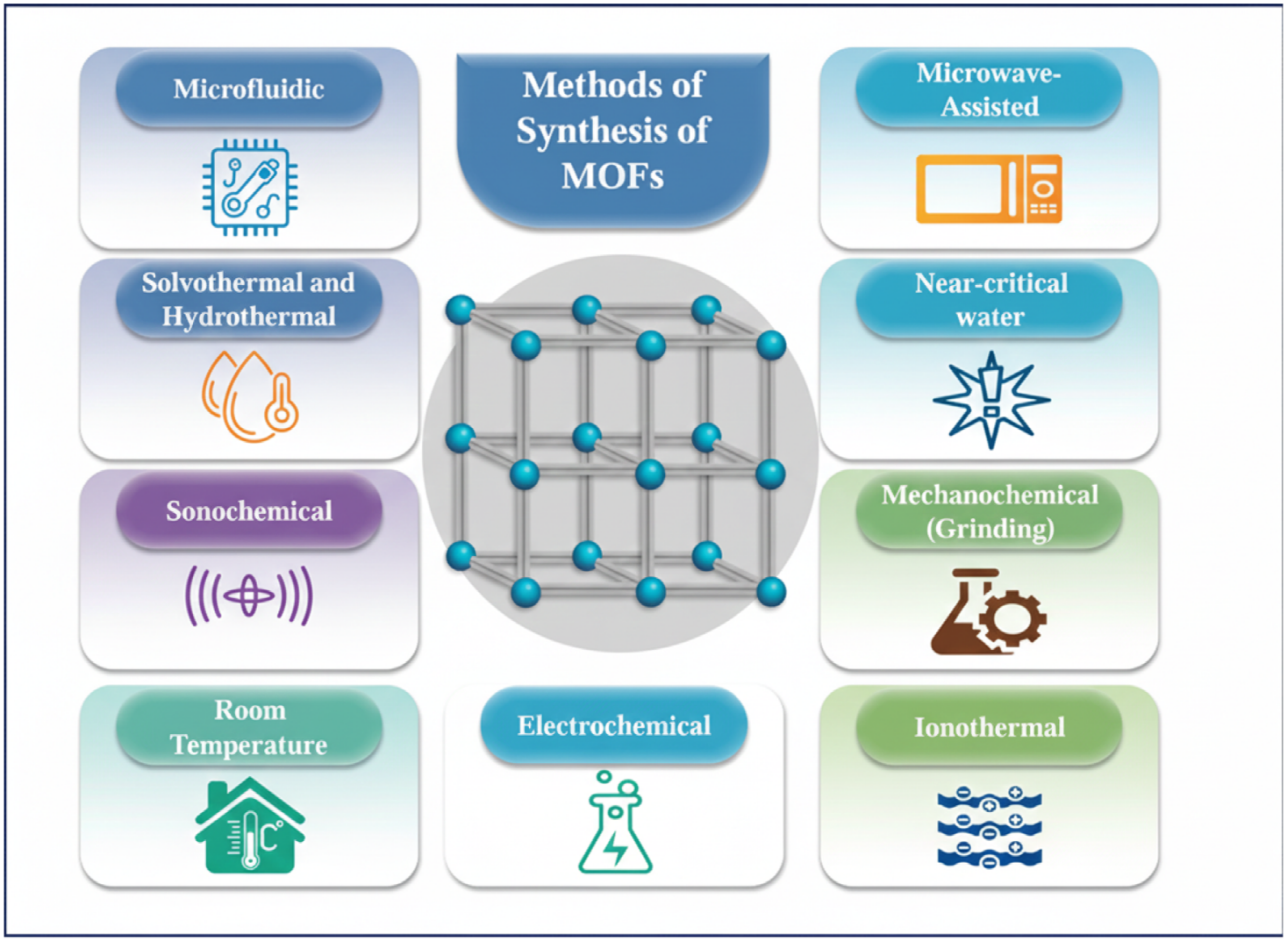
Summary of the main synthesis methods for MOFs.

The selection of metal salts, ligands, and solvents plays also a crucial role in the design and synthesis of MOFs [17, 18]. Metal nodes, which can be single ions or clusters, serve as coordination centers linking organic ligands into extended porous structures, and their choice strongly influences the structural, physical, chemical, and biological properties of the resulting MOFs. Commonly employed metals include first‐row transition metal ions, alkaline earth metals, and lanthanides due to their diverse coordination numbers, oxidation states, and geometries. Ligands critically determine the architecture, porosity, stability, and functionality of MOFs. The commonly used ligands include carboxylates, imidazolates, phosphonates, pyrazoles, and triazoles. The selection of ligands enables MOFs to be tailored for specific applications, including gas storage, catalysis, drug delivery, and sensing. Solvents not only provide the medium for reactant dissolution and interaction but also significantly influence crystallization, morphology, and product properties. Frequently used solvents include water, methanol, ethanol, DMF, DMA, and chloroform, with acids or bases sometimes added to enhance crystal quality and growth rates [19, 20, 21, 22]. Critical factors in solvent selection include solubility of the reactants, saturation vapor pressure, chemical compatibility, and environmental and safety considerations, with greener solvents such as water and ethanol preferred for their lower toxicity and reduced environmental impact. Deep eutectic solvents (DESs) can also influence MOF structures by modifying crystal morphology, controlling pore size, and enhancing framework stability. DESs consist of a hydrogen‐bond donor and acceptor and exhibit low toxicity, biodegradability, tunable properties, and the ability to dissolve diverse compounds [23, 24]. Figure 1 summarizes all methods for the synthesis of MOFs.

#### 1.1.1 Solvothermal and Hydrothermal Synthesis

Solvothermal synthesis remains one of the most widely employed methods for MOF fabrication. It involves dissolving metal salts and organic linkers in an appropriate solvent, followed by heating the mixture in a sealed autoclave at elevated pressure and temperatures (typically 80–250°C) [25, 26]. The method promotes controlled nucleation and growth of highly crystalline frameworks. Reaction time, solvent composition, and temperature directly influence the morphology and particle size. Solvothermal nanoparticle synthesis typically involves using diluted bulk‐reaction conditions to slow the reaction rate and promote uniform nanoparticle growth. The solvothermal method presents several drawbacks, including the high cost of autoclaves, difficulty in monitoring crystal growth, long reaction times that may extend over several days or even weeks, and the use of hazardous organic solvents in the reaction mixture [27, 28]. Zhu et al. prepared ZIF67@Ni(OH)_2_ composites via a solvothermal method, in which a Ni(OH)_2_ layer was grown on the surface of zeolitic imidazolate framework‐67 (ZIF‐67) under controlled water‐to‐methanol volume ratios [29]. Among the resulting series, the ZIF67@Ni(OH)_2_‐(1:1) sample retained the framework structure better than those synthesized in pure water or methanol and demonstrated excellent ethanol oxidation reaction (EOR) performance, achieving 10 mA cm^−2^ at + 1.34 V, long‐term stability exceeding 40 h, a high Faradaic efficiency of 97.1 %, and an ethanol‐to‐acetate conversion rate of 98.6 %.For synthesis, 80 mg of the Ni‐ZIF‐67 precursor, 4 mmol of NH_4_F, 20 mmol of urea, and 1 mmol of Ni(NO_3_)_2_ were dissolved in 40 mL of a 1:1 water–methanol mixture and stirred magnetically for 30 min. The solution was then transferred to a Teflon‐lined stainless‐steel autoclave and heated at 120°C for 6 h. The resulting precipitates were collected by centrifugation, washed three times with water and absolute ethanol, and dried overnight at 60°C. Deng et al. synthesized MOF‐1 using a solvothermal method. Briefly, 0.02 mmol of H_2_FIA, 0.02 mmol of 1,2‐BPYH, and 0.04 mmol of Cd(NO_3_)_2_·4H_2_O were dissolved in 1 mL of DMF/H_2_O (3:2, v/v). The mixture was sonicated for 15 min and then transferred into a 25 mL Teflon‐lined stainless‐steel autoclave. The reaction was maintained at 100°C for 96 h. After naturally cooling to room temperature, the resulting product was filtered and thoroughly washed to obtain yellow block crystals of MOF‐1. The synthesized material was subsequently utilized as a fluorescent sensor for the detection of chlortetracycline and as an adsorbent for Congo red in aqueous environments [30].

Controlling MOF particle size and morphology can be achieved by incorporating modulating agents typically monocarboxylic acids such as acetic acid, that compete with organic linkers for coordination sites on metal centers. The concentration of modulators affects the process in two ways. At low concentrations, the modulator increases the amount of deprotonated ligands, promoting the formation of nucleation centers and resulting in smaller particles. At high modulator concentrations, the likelihood of particle agglomeration increases, leading to the formation of larger aggregates due to more frequent crystal collisions. Surfactants are another class of modulators. Surfactants can stabilize nanosized droplets by forming a protective layer at the interface between two immiscible phases. In such systems, the size and shape of MOF can be controlled by adjusting surfactant‐to‐solvent ratios.

#### 1.1.2 Room Temperature Synthesis

This approach represents a distinct subset of solvothermal synthesis methods in which no external heating is required for the formation or crystallization of MOFs. Instead, the reaction between organic ligands and metal ions takes place at ambient temperature, allowing MOF crystallization under mild conditions.

During the synthesis, organic bases such as triethylamine are typically employed to deprotonate the ligands, facilitating coordination with the metal ions and subsequent MOF formation as a solid precipitate. This method is often favored due to its simplicity, energy efficiency, and high yields, as it eliminates the need for elevated temperatures or pressure [31, 32]. Xiang et al. synthesized Fe/Cu‐based MOFs (Fe/Cu‐MOFs) at room temperature over 24 h, which exhibited high stability and strong adsorption capacity for efficient tetracycline removal. The MOFs were prepared by dissolving varying FeSO_4_ · 7H_2_O:Cu(CH_3_COO)_2_ molar ratios of 1:0, 2:1, 1:1, 1:2, and 0:1 in deionized water Solution A. was added to a vigorously stirred solution of in 20 mL of water to form solution B. Solution B (6.67 mmol of H_3_BTC and 20 mmol NaOH) was then gradually added to solution A under magnetic stirring for 15 min, after which the mixture was stirred continuously at room temperature for 24 h. The resulting precipitate was collected by centrifugation, washed thoroughly with deionized water and ethanol, and dried under vacuum at 60°C for 12 h to obtain the final Fe/Cu‐MOF products [33].

However, the room temperature synthesis can be relatively slow, often requiring several hours to days for complete crystallization [34]. Additionally, some reactions must still be conducted under potentially hazardous conditions, utilizing solvents such as dimethylformamide (DMF) and acid modulators like hydrofluoric acid (HF) to promote framework formation and stability.

#### 1.1.3 Ionothermal Synthesis

The ionothermal approach utilizes low‐volatility ionic liquids as both eco‐friendly solvents and structure‐directing agents, effectively dissolving the precursors and facilitating the synthesis of MOFs with improved properties. Owing to their unique characteristics such as low vapor pressure, high solubility for organic compounds, exceptional thermal stability, broad liquid range, and nonflammability, ionic liquids represent a sustainable alternative for producing MOFs and other materials like zeolites and chalcogenides. Furthermore, by carefully selecting the cations and anions of the ionic liquids, this method allows the creation of diverse reaction environments, enabling the tailored synthesis of various MOFs with specific structural and functional features [35, 36, 37]. Kuchekar et al. developed MIL‐101 via an iono‐hydrothermal method. MIL‐101(Cr) was prepared using aqueous piperazinium dihydrogen sulfate ([H_2_Pi][HSO_4_]_2_) as a novel solvent system. Piperazine is a six‐membered heterocyclic amine containing two basic nitrogen atoms and is the third most commonly used N‐heterocycle in small drug molecules [38]. Recently, piperazine‐based ILs, including [H_2_Pi][HSO_4_]_2_ and piperazinium dihydrogen phosphate ([H_2_Pi][H_2_PO_4_]_2_), have gained attention as green solvents and catalysts for organic reactions, offering safer alternatives to corrosive mineral acids (HF, HNO_3_, HCl, and H_2_SO_4_) due to their high thermal stability, viscosity, ionic conductivity, and stable solid crystalline appearance. Notably, the optimal PZ‐15 % IL‐MIL‐101(Cr) synthesized at 7 h achieved an 89 % yield, a 35 % increase in CO_2_ adsorption capacity, and a 283 % enhancement in CO_2_/N_2_ selectivity, attributed to its high surface area (3671 m^2^/g) and large pore volume (2.74 cm^3^/g) [39].

The ionothermal method overcomes the limitations of the hydrothermal approach, such as the poor solubility of inorganic precursors and hydrogen‐bonding interference when water is used as a solvent. However, it also has drawbacks, including the difficulty of removing residual cations that remain trapped within the pores of the resulting MOFs.

#### 1.1.4 Microwave‐Assisted Synthesis

Microwave‐assisted synthesis offers a rapid and energy‐efficient approach to MOF preparation by ensuring uniform internal heating through interaction of microwave radiation with dipoles and ions in the reaction mixture. Microwaves are electromagnetic waves with frequencies ranging from 300 MHz to 300 GHz that can accelerate chemical reactions. They interact directly with reactant molecules and solvents by polarizing and coupling with them, inducing rapid rotation of polar and/or magnetic species in the reaction mixture. This enhanced molecular motion increases collision frequency, and the oscillating electric and magnetic fields of the microwaves generate localized molecular‐level heating, thereby accelerating reaction kinetics [40, 41].

This technique significantly accelerates nucleation and crystal growth up to 30 times faster than conventional heating, yielding particles with a narrow size distribution and enhanced crystallinity. The particle size can be finely tuned by controlling irradiation time, temperature, and precursor concentration. Morsi M. Mahmoud synthesized MOF‐801 via a microwave‐assisted method using zirconyl chloride octahydrate (ZrOCl_2_·8H_2_O) and fumaric acid. Specifically, 0.58 g of fumaric acid (5 mmol) and 1.6 g of ZrOCl_2_·8H_2_O (5 mmol) were dissolved in a solvent mixture of 20 mL DMF and 7 mL formic acid. The resulting solution was transferred into a 100 mL closed microwave Teflon (PTFE‐TFM) vessel capable of withstanding pressures up to 40 bar. The mixture was then directly heated using microwave irradiation to 110°C at a ramp rate of 50°C/min for 45 s to yield MOF‐801 [42].

#### 1.1.5 Ultrasound‐Assisted (Sonochemical) Synthesis

Ultrasound irradiation generates localized high temperatures (∼5000 K) and pressures (∼1000 bar) through the collapse of microbubbles, creating hot spots that drive rapid nucleation and crystallization. This sonochemical synthesis enables faster reaction rates and often produces smaller and more uniform nanoparticles than conventional or microwave methods. Additionally, sonication can break up agglomerated particles, improving dispersion and colloidal stability [38, 43]. Sonochemical synthesis has certain limitations, including a restricted heating depth and low product yield, which hinder its applicability for large‐scale MOF production.

#### 1.1.6 Electrochemical Synthesis

Electrochemical methods represent an alternative route that eliminates the need for high temperatures or long reaction times. In this approach, the metal ions are generated in situ from anodic dissolution of a metal electrode, while organic linkers are deprotonated at the cathode. The size of the resulting MOFs can be controlled by adjusting the applied voltage or altering the solvent ratios. Electrochemical synthesis also has several drawbacks, including low productivity and unsuitability for large‐scale production, primarily due to the requirement for bulky electrochemical setups and large‐area metal electrodes [44, 45, 46]. Lian et al. synthesized stable ZIF‐8 membranes on commercial ceramic supports using an electrochemical approach for efficient propylene/propane separation. The α‐alumina support was initially polished with sandpaper to promote uniform and rapid nucleation of ZIF‐8 crystals on its surface. The microstructure of the ZIF‐8 membranes was optimized by varying the metal ion‐to‐ligand ratio and the growth time. The growth mechanism on the ceramic support was investigated using linear sweep voltammetry and cyclic voltammetry. Ultimately, the limitations of the alumina support were overcome, enabling the direct synthesis of an ultrathin ZIF‐8 membrane (∼460 nm) on an unmodified porous alumina substrate, which exhibited excellent C_3_H_6_/C_3_H_8_ separation performance [47].

#### 1.1.7 Mechnochemical (Grinding) Synthesis

Mechanochemical synthesis, including liquid‐assisted grinding, involves the reaction of solid precursors in the presence of catalytic amounts of solvent under mechanical force such as ball milling). This solvent‐free or low‐solvent approach is environmentally friendly and allows rapid MOF formation at ambient conditions [48, 49, 50]. Perales et al. prepared a Zr‐based MOF (Zr‐MOF/2@RL) via a continuous‐flow mechanochemical approach using Zr‐methacrylate clusters as precursors. The MOF was synthesized in a Retsch PM100 ball mill equipped with a 125 mL reaction chamber and eighteen 10 mm stainless steel balls, operated at 200 rpm for 140 min. Zr‐benzoate and Zr‐methacrylate clusters, together with the organic linker BDC (0.102 mol), were added in a stoichiometric ratio of 1:6 (metal cluster:linker), along with 2.74 mL of methanol to form a paste. The resulting material was washed with solvent and filtered under vacuum. The prepared Zr‐MOF successfully removed 4‐nitrophenol (4‐NP) at room temperature, achieving optimal removal in 14 min—30 % faster than MOFs synthesized using a conventional method [51]. Liu et al. synthesized AgNPs@POMOF using a ball‐milling‐assisted approach for electrochemical sensing of heavy metals and food additives. Initially, 0.5 g of the crystals were ground into a fine powder using a ball mill. The resulting powder was dispersed in 10 mL of methanol to form a suspension, which was maintained in a 40°C oil bath under constant temperature. Subsequently, an aqueous AgNO_3_ solution (0.011 g AgNO_3_ dissolved in 4 mL of water) was added dropwise to the suspension under continuous stirring and allowed to react for 20 min. After the addition was completed, the mixture was further stirred for 10 h. Thereafter, a NaBH_4_ solution (0.011 g NaBH_4_ dissolved in 4 mL of water) was added, and the reaction was stirred continuously for an additional 10 h. The resulting solid product was collected by centrifugation and washed three times with anhydrous methanol. Finally, the product was dried in a vacuum oven at 80°C for 5 h, yielding 0.426 g (85% yield) of a brown–black powder [52].

Mechanochemical synthesis has certain limitations, including the difficulty of precisely controlling reaction conditions, the need for specialized grinders or mills, high energy consumption, low work efficiency, and the potential introduction of impurities from the milling equipment.

#### 1.1.8 Microfluidic Synthesis

Microfluidic reactors provide precise control over temperature, residence time, and reagent mixing, enabling uniform nucleation and growth of nanosized MOFs. In a microfluidic reactor, reaction solutions are pushed through narrow channels either within tubing or on a chip measuring just a few tens of micrometers in diameter. As the mixture flows through the reactor, it passes through heated sections. The small channel size allows rapid heat transfer, providing precise control over nucleation and crystal growth, and allowing reactions to occur within seconds up to 400 times faster than conventional solvothermal methods [53, 54].

#### 1.1.9 Near‐Critical Water Synthesis

Near‐critical water synthesis is an emerging technique that utilizes high‐temperature water as a reaction medium. This approach has been explored for applications including organic reactions, waste treatment, and nanoparticle formation. The method takes advantage of the unique properties of water as it nears its critical point. Specifically, the dielectric constant of water drops to values similar to those of nonpolar solvents, enhancing the solubility of organic compounds, such as the ligands commonly used in MOF synthesis [55]. The ionic product can increase by up to three orders of magnitude, reaching a maximum around 280°C. This creates a solvent environment with simultaneously high concentrations of H^+^ and OH^‐^ ions, enhancing reaction rates. Additionally, the water can be reused in subsequent reactions without prior purification [56, 57]. Ibarra et al. reported the synthesis of a zinc‐based MOF, using near‐critical water (300°C) as the reaction medium. The synthesis was conducted in a stainless‐steel high‐pressure batch reactor with an internal volume of ≈10 mL. The reactor was capable of operating up to 60 MPa at 400°C and was placed in an aluminium heating block surrounded by an electric ring furnace. For the reaction, Zn(NO_3_)_2_ · 6H_2_O (110 mg), 1,2,4,5‐tetrakis(4‐carboxyphenyl)benzene (H_4_L) (67 mg), and water (5 mL) were introduced into the reactor. The mixture was heated to 300°C at a rate of 45°C h^−1^ and maintained at this temperature for 48 h. During the reaction, the pressure inside the reactor reached 80 ± 2 bar at 300°C. After completion, the reactor was cooled to room temperature at a rate of 25°C h^−1^. The process yielded brown plate‐shaped single crystals, which were collected by filtration (78% yield) [56].

### 1.2 Defects Engineering

Traditionally, structural regularity and high crystallinity were considered ideal features of MOFs. However, recent studies have revealed that the intentional introduction and control of structural defects such as ligand deficiencies, metal node vacancies, or dislocations—are not mere flaws but can be strategically exploited to enhance material performance. In MOFs, lattice defects commonly appear as missing linkers or missing clusters, and their type and density can be finely tuned through both synthetic and postsynthetic approaches. This modulation creates a continuum of defectivity, resulting in diverse physicochemical properties and functionalities [58].

The emerging subfield of defect engineering focuses on the deliberate creation and manipulation of such imperfections to produce MOFs with tailored characteristics. Point defects, including missing linker and missing cluster defects, have shifted the conventional perception of MOFs by demonstrating that deviations from perfect crystallinity can actually enhance material performance. For instance, missing linker defects often generate additional active sites, which can improve adsorption capacities, catalytic activity, and overall reactivity [59, 60].

Furthermore, defect engineering allows for the modification of pore structures, thereby enhancing molecular sieving and selective separation capabilities. This has significant implications for applications such as gas separation, catalysis, and sensing, where performance can surpass that of defect‐free frameworks.

### 1.3 Characterization of Metal–Organic Frameworks

**FIGURE 2:**
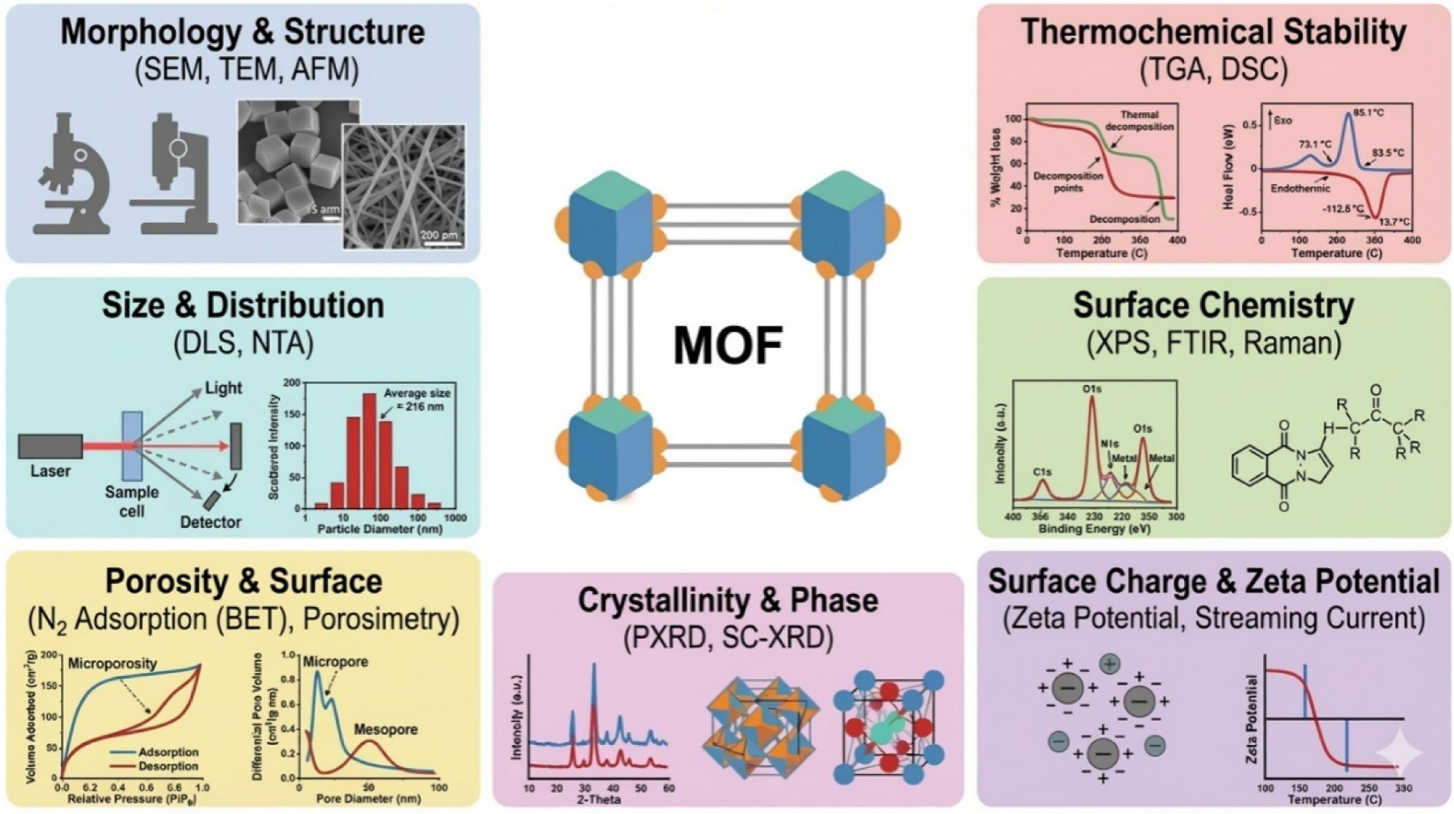
Common techniques used for the characterization of MOF.

Characterization of MOFs is essential for understanding their structure, morphology, size, porosity, and physicochemical properties, which directly affect their performance in catalysis, adsorption, and sensing applications [61]. The commonly employed techniques for MOF characterization include microscopic, spectroscopic, and diffraction‐based methods Figure 2.

#### 1.3.1 Morphological and Structural Characterization

The morphology and surface topography of MOFs can be examined using scanning electron microscopy (SEM) and transmission electron microscopy (TEM).

SEM provides information about particle size, surface texture, and morphology at Nanometer resolution, though only surface features are visible. Energy‐dispersive X‐ray spectroscopy coupled with SEM enables elemental mapping and confirmation of metal and ligand distribution [62, 63].

TEM, on the other hand, offers subnanometer resolution and allows visualization of internal structures, shape, and crystal domains, making it suitable for determining the physical dimensions and orientation of nanoparticles. High‐resolution transmission electron microscopy (HR‐TEM) can be employed to verify crystallinity and visualize lattice fringes at the nanoscale. TEM uses a high‐energy electron beam, which can potentially damage the sample and alter the particle dimensions, and it requires a high‐vacuum environment. Consequently, it is not suitable for fragile nanoparticles that need to remain in a solvent. Unlike SEM, conventional TEM does not require a conductive coating, but it interacts directly with the fixed sample, which is often stained with vacuum‐deposited platinum to enhance imaging contrast. To avoid sample fixation, cryo‐TEM can be used, where the sample is rapidly frozen to preserve its native structure [64].

Atomic force microscopy (AFM) provides 3D topographical data with up to 0.1 nm resolution under ambient conditions without the need for sample preparation such as filtration or coating, which is required for SEM. Measurements can be performed on dry samples or in solution using tapping mode. Like SEM and TEM, AFM is a valuable tool for assessing the shape, structure, and size of nanoparticles. Its major advantage lies in its ability to image biomaterials and their interactions, including processes such as aggregation in aqueous environments [65]. However, AFM tends to overestimate lateral dimensions due to the size of the cantilever tip relative to the nanoparticles. AFM images typically cover areas of several hundred micrometers, and data acquisition can take several minutes depending on the imaging mode, making it less suitable for high‐throughput nanoparticle characterization [66].

#### 1.3.2 Particle Size and Distribution

The particle size and size distribution of MOFs influence their surface area, stability, and catalytic performance. Dynamic light scattering (DLS) and static light scattering are used to determine the hydrodynamic diameter of MOF nanoparticles in suspension. For nanoparticles larger than 30 nm, complementary methods such as nanoparticle tracking analysis (NTA; 30–1000 nm) and fluorescence‐activated nanoparticle sorting (>100 nm) are increasingly employed. NTA and differential centrifugal sedimentation provide high‐resolution measurements of size distributions in polydisperse samples [67, 68, 69].

#### 1.3.3 Crystallinity and Phase Identification

The crystalline nature of MOFs is crucial to their framework integrity and porosity. Single‐crystal X‐ray diffraction (SCXRD) provides detailed atomic arrangements within a crystal, offering precise information on unit cell geometry and stability. However, SCXRD requires large, high‐quality crystals (>100 μm) and can be time‐consuming. When suitable single crystals are unavailable, powder X‐ray diffraction (PXRD) is used instead, which provides structural information from polycrystalline samples in powdered samples. PXRD can identify crystal phases, measure lattice strain, determine crystallite size via the Scherrer equation, and monitor changes in morphology [70, 71].

In cases involving nanoscale crystals (<10 nm), small‐angle X‐ray scattering (SAXS) becomes valuable for analyzing pore structures, particle size distributions, and hierarchical organization at Nanometer scales. SAXS operates at low scattering angles (0.1°–10°) and complements PXRD by probing smaller structural features. In general, PXRD is used for wide‐angle analysis of large crystalline structures (>100 nm), whereas SAXS is applied to characterize nanostructures and fine morphological details.

#### 1.3.4 Porosity and Surface Area

The porosity of MOFs determines their adsorption and diffusion properties. Based on pore dimensions, MOFs are classified as microporous (<2 nm), mesoporous (2–50 nm), or macroporous (>50 nm) [72, 73]. Gas adsorption–desorption isotherms are employed to estimate surface area and pore volume. Nitrogen is often used as a small, inert gas at a temperature near its boiling point of 77 K, provided that the pores are larger than 0.7 nm. For smaller pores (<0.7 nm), argon adsorption at 87.3 K is preferred due to its ability to access narrower pores more effectively. Nitrogen or argon measurements can typically detect surface areas as low as 0.5 m^2^ g^−1^, while krypton adsorption at 77 K is employed for materials with extremely low surface areas (<0.05 m^2^ g^−1^). For pores smaller than 0.45 nm, carbon dioxide adsorption at 273 K is used, and CO_2_ adsorption at 298 K is often applied to determine the total pore volume of ultramicroporous materials [74].

The profiles of the adsorption and desorption isotherms offer insights into the porosity of the material. The intrinsic porosity is influenced by synthesis conditions such as solvent type, temperature, use of modulators or secondary metals, and the activation method employed. Variations in these factors can lead to significant changes in surface area and pore structure, impacting the performance of the final material [66].

#### 1.3.5 Thermal and Chemical Stability

Thermogravimetric analysis is used to examine the thermal stability and decomposition behavior of MOFs. The temperature‐dependent weight loss profile offers valuable information about the removal of guest molecules, framework decomposition, and residual metal oxide formation. Understanding the thermal stability of a MOF is crucial for determining appropriate synthesis and activation conditions, particularly when designing materials for catalytic or environmental applications under elevated temperatures [75].

#### 1.3.6 Surface Chemistry

Fourier transform infrared spectroscopy (FTIR) spectroscopy serves as a powerful tool to identify the functional groups present in MOFs and assess the nature of metal–ligand coordination. Characteristic vibrational bands corresponding to –COOH, –OH, –NH_2_, –NO, and –CN groups provide information about the bonding environment and framework stability. FTIR's versatility, allowing analysis of powders, thin films, and fully fabricated devices, makes it a standard technique for verifying chemical integrity and functionalization of MOFs. Furthermore, in situ FTIR techniques are increasingly emphasized for real‐time monitoring of synthesis and postsynthetic modification (PSM) processes [76]. X‐ray photoelectron spectroscopy (XPS)PS can also reveal elemental composition identifying metals, nonmetals, and other elements that are present on the nanoparticle surface. It also determines the oxidation states of metal centers or the presence of functional groups on linkers [69, 77].

#### 1.3.7 Surface Charge and Zeta Potential

The surface charge of MOFs plays a crucial role in determining their colloidal stability, interparticle interactions, and adsorption behavior toward charged species. Characterizing the surface charge provides insights into electrostatic interactions between MOF particles and their surrounding environment, which directly influence performance in aqueous‐phase catalysis, separation, and sensing applications. The surface charge of MOFs depends strongly on pH, ionic strength, and surface functional groups introduced during synthesis or PSM [78, 79].

The zeta potential is the most widely used parameter to evaluate surface charge. It is determined by measuring the electrophoretic mobility of MOF particles in suspension using electrophoretic light scattering (ELS). ELS measures the motion of charged nanoparticles under the influence of an applied electric field. Similar to DLS, a laser beam is directed at the particle suspension, but in ELS, an external electric field is applied. The particles move in response to the field, and the scattered light from these moving particles experiences a frequency shift (Doppler shift). This shift is analyzed to calculate the velocity of the particles, which is then used to determine their zeta potential. ELS is commonly performed alongside DLS due to their similar experimental setup.

## 2 Postsynthetic Modification of MOFs

PSM represents one of the most straightforward and versatile approaches to introduce new functionalities into MOFs while maintaining their intrinsic features such as crystallinity, structural integrity, and porosity. Although early examples of postsynthetic transformation of coordination networks were reported as early as 1999, the term PSM was formally established in 2007. Since then, the chemical toolbox available for MOF functionalization has expanded substantially, enabling tailored modifications for diverse applications in catalysis, sensing, gas storage, and drug delivery [80, 81, 82, 83].

PSM offers a unique advantage over direct synthesis by allowing the incorporation of reactive or delicate functional groups under mild conditions, thus avoiding the harsh solvothermal environments that typically restrict the inclusion of sensitive moieties. Through PSM, the physical and chemical properties of MOFs such as surface polarity, catalytic activity, hydrophobicity/hydrophilicity, and adsorption selectivity can be finely tuned without compromising their overall architecture [84, 85, 86, 87, 88].

Depending on the chemical nature of the transformation and the part of the framework involved, PSM strategies can be categorized into four principal pathways:

### 2.1 Postsynthetic Ligand Modification

This pathway focuses on exploiting the reactive organic linkers embedded within the MOF framework using well‐established organic chemistry reactions. Through this approach, functional groups can be covalently anchored to the framework (termed covalent or dative PSM), or existing labile substituents can be transformed via thermal or photochemical reactions (PS transformations). Such modifications enable the incorporation of catalytic sites, sensing moieties, or hydrophilic/hydrophobic groups directly into the framework, thereby enhancing its performance in specific applications [80, 81].

### 2.2 Postsynthetic Ligand Exchange or Insertion

An alternative strategy involves partial replacement or insertion of linkers within the MOF lattice. This process takes advantage of the dynamic nature of the metal–carboxylate (M–COO) coordination bonds, allowing selective exchange of existing linkers with new ones. When the incoming linker does not substitute an existing one but instead occupies vacant or defective sites, the process is referred to as ligand insertion. PSLE and PSLI are particularly useful for modulating pore size, chemical environment, and framework composition, leading to the development of mixed‐linker or hierarchical MOFs [82, 84, 85]. Bingel et al. investigated the influence of postsynthetic ligand exchange (PSLE) on the flexible MOF ZIF‐7, focusing on its gate‐opening pressure and CO_2_/CH_4_ separation performance. Nitrogen‐containing ligands—namely 2‐aminobenzimidazole, benzotriazole, and 5‐azabenzimidazole—were incorporated into ZIF‐7 via PSLE. These ligands introduce nitrogen atoms either within a heterocyclic ring or as attached functional groups. The modification was designed to enhance the framework's affinity for CO_2_ over CH_4_, thereby improving selectivity in biogas separation applications. Zou et al. developed a new MOF, LeZIF‐8‐PA, via ligand exchange between ZIF‐8 and phthalic acid for enzyme immobilization. Lipase immobilized on ZIF‐8 exhibited poor stability during the methanolysis of soybean oil for biodiesel production. In contrast, LeZIF‐8‐PA demonstrated enhanced stability in both pure oleic acid and aqueous environments. Furthermore, lipase immobilized on LeZIF‐8‐PA_0_._5_ showed higher specific activity and improved reusability compared to ZIF‐8, making it more effective for biodiesel production [89].

### 2.3 Postsynthetic Metalation

PSMet techniques are designed to introduce additional metal ions or clusters into preformed MOFs in a controlled manner. Depending on the reaction target, this can occur through:

Inorganic PSMet, where metal exchange or insertion takes place at the secondary building units; Shi et al. successfully synthesized a bimetallic Zr/Hf‐based UiO‐66 MOF from a preformed Zr‐UiO‐66 through a mechanochemical metal‐exchange process, a transformation that is typically difficult to accomplish under conventional solvothermal conditions. The resulting Zr/Hf‐UiO‐66 framework demonstrated enhanced acidic properties and remarkable catalytic performance in the esterification of levulinic acid, highlighting the effectiveness of the mechanochemical approach for postsynthetic metal incorporation [90].

Dative PSMet, where new metal centers coordinate to donor atoms (e.g., N, O, or S) located on the linkers Lim et al. developed a Pd(II)‐incorporated Ni‐MOF‐74 catalyst by employing aminosalicylic acid as a functionalized fragmentary organic ligand. The introduction of amino groups into the MOF framework was achieved through direct solvothermal synthesis using n‐aminosalicylic acid (H_2_n‐aSA), yielding an amine‐functionalized MOF (DEMOF‐I) that preserved its crystallinity and permanent porosity despite the presence of framework defects.

Subsequent covalent PSM converted the amino groups of DEMOF‐I into iminopyridine moieties via condensation with pyridine aldehyde, thereby generating metal‐binding sites. A following postsynthetic metalation step introduced Pd(II) ions, producing the Pd(II)‐incorporated Ni‐MOF‐74 catalyst. This catalyst demonstrated highly efficient, size‐selective, and recyclable catalytic activity in the Suzuki–Miyaura cross‐coupling reaction between phenylboronic acid and various aryl bromides [91].

Encapsulation or Ion Exchange, where encapsulate metallic species (either in the form of free ions or structured into INPs, polyoxometalates, etc.) are confined within the porous cavities of the framework. Wang et al. reported the encapsulation of MAg_24_ (M = Ag, Pd, Pt, or Au) nanoclusters (NCs) within the MOF UiO‐66‐NH_2_, forming MAg_24_@UiO‐66‐NH_2_ composites. Among these, AuAg_24_@UiO‐66‐NH_2_ exhibited the highest photocatalytic activity, outperforming all other counterparts and showing excellent recyclability in hydrogen evolution reactions [92].

### 2.4 Postsynthetic Macroscopic Etching

Unlike the previous methods that target chemical functionalities, PSEtch focuses on modifying the morphological and surface characteristics of MOF crystals. Controlled etching treatments can reshape crystal surfaces, alter particle size, or introduce hierarchical mesoporosity. Such structural tailoring can significantly improve mass transport, enhance catalytic accessibility, and expose additional active sites without altering the intrinsic framework chemistry. Zheng et al. introduced an alkali etching strategy for the modification of MOF‐derived spinel CoMn_2_O_4_, providing a synergistic approach that combines nanoparticle disaggregation with Mn–O bond modulation. During the etching process, hydroxide ions (OH^‐^) react with the metal surface, producing hydroxyl species that increase surface charge density and prevent nanoparticle agglomeration through electrostatic repulsion. Simultaneously, sodium ion (Na^+^) doping alters the local Mn–O bonding environment by enhancing the electron cloud density around Mn atoms. This results in a bond elongation from 2.06 Å to 2.18 Å and a bond strength reduction from 250.41 N·m^−1^ to 245.68 N·m^−1^ compared to the undoped MOF‐derived material. Moreover, NaOH etching induces crystal reconstruction and creates surface defects, further adjusting the Mn–O coordination and improving nanoparticle dispersion [93].

This structural disaggregation significantly increases the specific surface area and exposes a greater number of active sites, thereby enhancing the adsorption and activation of reactant molecules. The formation of longer and weaker Mn–O bonds also facilitates lattice oxygen release and oxygen vacancy generation, promoting the formation of reactive oxygen species (ROS) crucial for catalytic activity.

Unlike traditional single‐parameter modification techniques, this dual modulation approach effectively bridges macroscopic structural dispersion with atomic‐level electronic tuning, simultaneously mitigating nanoparticle aggregation and improving the intrinsic catalytic performance of the material. SEM and HRTEM analyses confirmed a distinct morphological transition—from densely packed particles in CoMn‐M to highly dispersed nanoparticles, corroborating the enhanced structural and electronic modulation achieved through Na^+^ doping and alkali etching.

## 3 Hybridization Strategies

**FIGURE 3:**
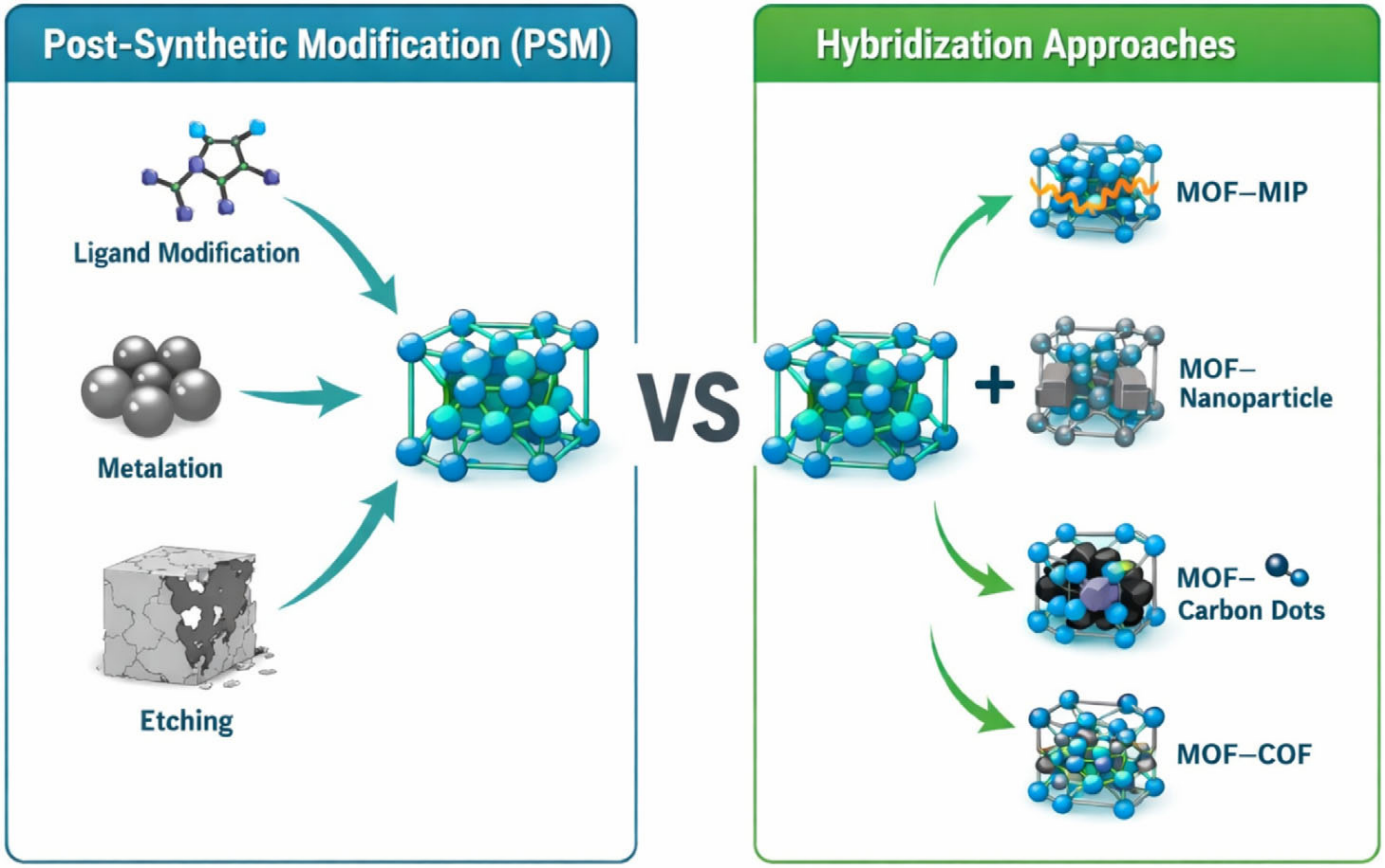
Schematic comparison of PSM and hybridization approaches for MOFs.

While PSM involves chemically or structurally altering a preformed MOF framework, hybridization strategies focus on combining MOFs with external materials to form composite or hybrid systems, as illustrated in Figure 3. Unlike PSM, which modifies the intrinsic properties of the MOF itself, hybridization leverages the synergistic integration of MOFs with polymers, nanoparticles, carbon‐based materials, or other frameworks to enhance stability, functionality, and performance in a broader range of applications [94, 95, 96].

There are three main ways to achieve MOF hybridization. The first is encapsulation, which involves introducing other materials into the pores of an existing MOF without forming direct covalent bonds. In this case, the notation A@B indicates that material A is encapsulated within material B. Encapsulation has been widely used to create MOF hybrids with improved stability, enhanced performance, and often new functionalities. The second approach is deposition, which typically produces membrane‐like materials by layering MOFs and other components. Deposition is similar to encapsulation because the added materials can adhere to the MOF surface, and the incorporated elements can be tailored for specific applications. In layer‐by‐layer deposition, the notation A|B|C represents materials A, B, and C assembled sequentially in a hybrid form. The third method is in situ growth, where MOF crystals are formed through solvothermal or hydrothermal reactions of metal salts and ligands in the presence of another material. The second material usually acts as a structure‐directing agent, guiding the oriented growth of MOF crystals. Hybrids obtained by in situ growth often exhibit ordered morphologies and hierarchical structures, which are beneficial for applications such as gas storage and small molecule separations. A MOF grown in situ on another material A is denoted as MOF@A [4].

In the next section, we will highlight remarkable examples of MOF hybrids and their synthesis strategies. The mechanisms by which MOF‐based hybrids enhance performance are summarized in Table 2.

**TABLE 2:** Mechanistic overview of MOF‐based hybrids.

MOF hybrid	Key improvement mechanism	Explanation of enhancement	Ref
**MOF–QDs (Quantum Dots)**	Enhanced electron transport and photocatalysis	QDs improve the conductivity of otherwise poorly conductive MOFs. Band alignment at the MOF–QD interface promotes effective charge separation, suppressing electron–hole recombination, while strong photoluminescence enables sensitive and selective fluorescence sensing. The composite also shows superior stability compared to individual components.	[97, 98]
**MOF–Nanozymes (NZs)**	Increased catalytic activity and substrate accessibility	The porous MOF architecture exposes more catalytically active sites and facilitates substrate diffusion. Synergistic interactions between MOF and NZ enhance catalytic efficiency. Mechanical stability and guest adsorption are improved compared to unmodified MOFs.	[99]
**MOF–MIPs**	Selective recognition and enhanced mechanical stability	MOFs provide tunable pore sizes and chemical functionality, improving accessibility to imprinted sites. Integration with MIPs increases specific surface area, enhances mechanical strength, and improves selective binding for target molecules.	[100]
**MOF–DESs**	Increased adsorption sites and interaction strength	DESs introduce additional adsorption sites and new functionalities, enabling noncovalent and electrostatic interactions critical for extraction. Synergistic interactions between DES and MOF enhance stability and adsorption capacity.	[23]
**MOF–MXenes**	Improved electrochemical performance	Synergistic interaction between MOF and MXene boosts conductivity and charge transfer. Unique hybrid morphologies lead to higher surface area, facilitating ion transport and enhancing electrochemical properties beyond individual components.	[101]
**MOF–COFs**	Enhanced porosity, mass transfer, and catalytic activity	Covalent integration creates core–shell MOF@COF structures with high surface area and ordered porosity. This improves mass transfer, accessibility to active sites, and molecular recognition, enhancing catalysis, sensing, and separation performance.	[102]

### 3.1 Hybrid with Quantum Dots

QDs are quasi‐zero‐dimensional (0D) fluorescent semiconductor nanocrystals, typically ranging from 1 to 10 nm in size and often spherical or nearly spherical in shape. Their unique size‐dependent electronic properties have driven rapid advancements in applications such as sensing, biological imaging, medical diagnostics, and catalysis. QDs are composed of aggregates of atoms and molecules at the nanoscale, with traditional QDs often made from Group II–VI and III–V elements, typically forming core–shell structures (e.g., CdSe/ZnS, InP/ZnS, CdTe/TiO_2_, PbS/ZnSe) [103]. The MOF@QDs composites exhibit numerous advantageous properties, including enhanced luminescence performance and good biocompatibility. Key benefits include: (1) improved conductivity, as most MOFs are poorly conductive but QDs enhance electron transport in the composite; (2) strong photoluminescence, enabling MOF@QDs to serve as highly sensitive and selective fluorescence sensing platforms; (3) effective charge separation at the QD–MOF interface due to band alignment, which suppresses electron–hole recombination and enhances photocatalytic performance; and (4) superior stability compared to the individual components.

Beyond these advantages, QDs in MOF@QDs composites can act as optical signal markers and reaction carriers. The high surface area of the composite increases the probability of molecular collisions, accelerating reaction rates and improving the overall sensitivity of sensing or catalytic systems.

Various strategies have been developed to construct MOF@QDs composites, with each method influencing the resulting properties, such as fluorescence, catalysis, or conductivity. The most established approaches are ‘Ship in the Bottle’ and ‘Bottle around Ship’, while other methods include photochemical deposition, physical mixing, direct surface functionalization, and several emerging techniques.

Ship in the Bottle SIB involves introducing QD precursors into the pores of a preformed MOF, where they grow in situ to form the composite. This method helps prevent QD aggregation and maintains a conformal MOF layer around the QDs, but it requires careful control of MOF stability, precursor compatibility, and synthesis conditions. Variations include solution permeation, chemical vapor infiltration, and double solution methods.

Bottle around Ship BAS, also known as the template strategy, begins with presynthesized QDs stabilized by surfactants or capping agents. MOF precursors are then assembled around the QDs to form the composite. This approach allows higher QD loading, reduces aggregation, and permits precise control over QD size and morphology.

Tran et al. investigated the encapsulation of InP/ZnS QDs using two different strategies: BAS and SIB approaches [104]. In the BAS method, the MOF was synthesized around the QDs. This process began with a ligand exchange step to disperse the QDs in the MOF synthesis solvent, DMF. A room‐temperature synthesis route was selected to ensure compatibility with the fragile InP nanoparticles and to avoid the strong acidity typically associated with conventional solvothermal methods. Triethylamine (Et_3_N) was used as a mild organic base to maintain a controlled environment.

In contrast, the SIB approach involved a modified version of the conventional MOF‐5 synthesis, where cetyltrimethylammonium bromide (CTAB) was introduced as a templating agent. CTAB has been shown to promote the formation of mesoporous MOF‐5 structures under solvothermal conditions. This modification allowed the QDs to be incorporated into the MOF framework through capillary action.

Photochemical Deposition is another strategy that uses light to induce in situ formation and deposition of QDs on the MOF surface. It is simple, cost‐effective, and operates under mild conditions, but requires QD precursors with suitable redox properties and is limited by light absorption efficiency. Physical Mixing is the simplest approach, involving presynthesized QDs and MOFs combined via nonspecific interactions, physisorption, or ultrasonic fusion. Electrospinning can be integrated with this method to produce MOF@QDs nanofibers with tunable diameters. Direct surface functionalization relies on functionalized QDs and MOFs with complementary ligands. Functional groups on QDs interact with MOF metal sites through in situ ligand exchange, allowing precise control over QD and MOF morphology. Other methods include electrochemical deposition, electrophoretic deposition, in situ encapsulation, calcination, and emerging strategies such as in situ stepwise growth and electrostatic assembly. These approaches expand the versatility of MOF@QDs composites for applications in sensing, catalysis, and optoelectronics [97, 98, 105].

### 3.2 Hybrid with Nanozymes

NZs are nanomaterial‐based artificial enzymes, which exhibit intrinsic enzyme‐like catalytic activities along with high chemical and thermal stability [106]. MOF@ NZs offer significant advantages because the porous architecture of MOFs increases the exposure of catalytically active sites. It also facilitates substrate diffusion and selective interactions, which improves overall catalytic performance. Recent studies have shown that MOFs and their derivatives exhibit superior catalytic activity. This is attributed to their hierarchical structures and the presence of multiple accessible active sites. Immobilizing NZs on MOF carriers enhances operational stability, reusability, and catalytic efficiency [99]. Several strategies are employed to fabricate MOF@ NZ composites. Surface adsorption is one of the simplest and most widely used methods, relying on nonspecific interactions such as hydrogen bonding, van der Waals forces, and electrostatic attraction to attach the NZ onto the MOF surface. However, this approach can suffer from NZ leaching during use and often exhibits low loading efficiency, limiting the amount of active enzyme or NZ immobilized. Covalent linkage offers a more robust alternative, forming strong chemical bonds between the NZ and functional groups on the MOF surface, enhancing stability and retention. Pore infiltration is another strategy, where NZs are embedded within the internal cavities of mesoporous MOFs, taking advantage of the high surface area and protective environment of the pores. Finally, de novo encapsulation involves the in situ growth of MOFs around the NZs, typically through processes such as coprecipitation, chemical deposition, microfluidics, or mechanochemical synthesis. This ‘one‐pot’ approach is independent of MOF pore size and structure, providing a versatile platform for various biomolecules. It offers multiple benefits, including tunable enzyme activity, enhanced stability, high catalytic efficiency, and improved sensitivity. Chen et al. synthesized CeCu‐MOFs with outstanding intrinsic peroxidase‐, oxidase‐, and catalase‐like catalytic activities through an environmentally friendly one‐step chemical deposition method. Furthermore, they developed a catalytic sensing platform based on the oxidase‐like activity of the CeCu‐MOFs for the quantitative detection of ascorbic acid (AA) with signal amplification capability [107].

### 3.3 Hybrid with DES

DES‐functionalized MOFs combine the remarkable properties of MOFs—such as high porosity, large surface area, and structural diversity with the solubilization capabilities of DESs. These composites exhibit enhanced mechanical strength, stability, and guest adsorption capacity compared to their unmodified counterparts, which is attributed to the synergistic interactions between the DES and MOF components. The incorporation of DESs can also increase the number of adsorption sites within MOFs or introduce new functionalities, facilitating noncovalent or electrostatic interactions that are crucial for the extraction process [23].

The wet impregnation method is the most commonly employed postsynthetic approach for incorporating DESs into MOFs. In this method, DESs are first dissolved in an appropriate inert solvent, such as acetone, methanol, or ethanol, to achieve optimal dispersion. MOF powder is then added to the solution and stirred at room temperature for several hours. After a homogeneous mixture is obtained, the solvent is evaporated, resulting in a powdered DES/MOF composite. This approach has been widely used to prepare DES/MOF composites for applications in adsorption, separation, and catalysis. Another approach is ligand functionalization that can involve attaching functional reagents, such as preprepared DESs, to the MOF ligands, exposing free amino or carboxyl groups. Subsequent acid–base condensation reactions integrate the DESs into the MOFs. To preserve the integrity of the functionalized MOF, ligand functionalization should be performed in a way that maintains the primary structure and overall shape of the parent material [108, 109].

### 3.4 Hybrid with Molecular Imprinted Polymers

Molecular imprinting technology (MIT) is recognized as a versatile and promising approach capable of selectively recognizing a wide range of biological and chemical molecules, including amino acids, proteins, nucleotide derivatives, pollutants, drugs, and food components [100, 110, 111]. Its applications extend to separation and purification processes, chemical sensing, catalysis, drug delivery, and biological systems involving antibodies and receptors. MIT relies on the formation of a complex between a target molecule (template) and a functional monomer. In the presence of a large excess of a cross‐linking agent, a 3D polymer network is formed. Once polymerization is complete, the template is removed, leaving behind recognition sites that are complementary in shape, size, and chemical functionality to the template molecule [112, 113].

The combination of molecularly imprinted polymers (MIPs) with MOFs is particularly attractive. MOFs offer tunable pore sizes by adjusting the lengths of organic linkers, and their chemical functionality can be modified by selecting different metal nodes and ligands during synthesis. Integrating MIPs with MOFs enhances accessibility to the imprinted sites while providing additional benefits, including improved mechanical stability and a high specific surface area [114]. MOF@MIP composites can be prepared using several strategies, each offering distinct advantages. In the bulk polymerization approach, template molecules and functional monomers are prepolymerized in a solvent, followed by the addition of crosslinkers and initiators to form a 3D polymer network. The resulting polymer is then dried, crushed, ground, and sieved into fine powder, and the template molecules are removed to create specific recognition sites. This method produces MOF@MIPs with excellent selectivity and recognition properties. Electropolymerization is another strategy, in which electroactive or electropolymerizable monomers, such as o‐phenylenediamine, pyrrole, or aniline, are deposited onto an electrode surface in the presence of the template under a suitable potential. This approach enables strong electrostatic interactions between the monomer and template, offers controlled film thickness, and enhances sensitivity due to the conducting polymer matrix. Incorporating MOFs in the monomer solution can further improve sensitivity and expand applications in sensing technologies. Surface molecular imprinting represents a third approach, where polymerization occurs directly on the surface of preformed MOFs. In this case, recognition sites are localized on the outermost layer, allowing rapid surface binding of target analytes. This technique provides faster binding kinetics, higher separation efficiency, and has been widely applied in catalysis, sensing, and biomedical fields. Standard MIP reagents are typically used for polymerization under conventional conditions, yielding MIP@MOF composites with accessible and highly selective recognition sites [112].

Han et al. employed surface polymerization to fabricate novel MIPs (MOF@DES‐MIPs) for the selective adsorption of bovine hemoglobin (BHb) [24]. In their design, modified MOF‐199 served as the substrate, BHbwas used as the template protein, and a DES composed of choline chloride and methacrylic acid acted as the functional monomer. The MOF substrate improved the accessibility of the imprinted sites, while the DES monomers, through multiple interactions with BHb, facilitated the formation of specific recognition cavities. The resulting imprinted polymer film exhibited high selectivity toward the target analyte. The study also evaluated the effects of BHb concentration and adsorption time on the performance of MOF@DES‐MIPs. The maximum adsorption capacity reached 151.28 mg g^−1^, with rapid adsorption equilibrium and excellent selectivity. This strategy offers a promising approach for the preparation of MIPs for biomacromolecules, and MOF@DES‐MIPs were successfully applied for selective recognition of BHb from a real bovine blood sample.

### 3.5 Hybrid with MXenes

MXenes are a new class of 2D transition metal carbides and nitrides. They represent an emerging family of advanced 2D materials with high electrical conductivity and rich surface chemistry [115]. The electrochemical performance of MOF@MXene hybrids is superior to that of either component alone due to their synergistic interaction. MOF‐derived materials combined with MXenes also show unique morphologies and excellent electrochemical properties [101, 116, 117, 118].

Lan et al. reported the synthesis of ultrasmall Pd nanocrystals embedded within Co‐based MOF‐decorated Ti_3_C_2_T_*x*_ MXene (Pd/MOF‐MX) nanoarchitectures using a controlled solvothermal method [119]. In this design, ultrathin MXene nanosheets combined with porous Co‐based MOFs form an efficient hybrid support, providing high electrical conductivity and large accessible surface areas. This structure not only enables precise size confinement and uniform distribution of Pd nanocrystals but also enhances their intrinsic catalytic performance through direct electronic interactions. Owing to strong synergistic effects, the Pd/MOF‐MX nanoarchitectures demonstrate significantly improved electrocatalytic activity for methanol oxidation, featuring a large electrochemically active surface area of 116.7 m^2^ g^−1^, a high mass activity of 1700.4 mA mg^−1^, and excellent long‐term stability. These properties outperform those of conventional Pd catalysts supported on carbon black, carbon nanotubes, graphene, and unmodified MXene matrices. Li et al. synthesized a UiO‐66‐NH2/MXene aerogel composite via a hydrothermal approach followed by freeze‐drying. This composite exhibited highly efficient and selective removal of U(VI) from aqueous solutions, achieving a removal efficiency greater than 96%. The UiO‐66‐NH_2_/MXene aerogel acts as both U(VI) adsorbent and capacitive deionization electrode. The removal rate of the composite can be up to 95% with the C_0_ 100 mg·L^−1^ at pH 6.0.The study highlights the potential of designing tailored MXene‐MOF composites for specific applications. The combination of the porous structure of MOFs with the conductive and mechanically robust properties of MXenes provides enhanced adsorption kinetics, selectivity, and stability [120].

### 3.6 Hybrid with COFs

Hybrid materials combining MOFs and COFs offer additional advantages by integrating the unique properties of both components. COFs possess highly ordered structures, and when covalently linked with MOFs in a core–shell configuration, MOF@COF composites are formed [121]. Nanoscale MOF@COF structures exhibit high porosity and large surface areas, providing abundant imprinting sites and improving mass transfer rates, which can be advantageous for applications such as catalysis, sensing, and molecular recognition [122].

In COF‐on‐MOF hybrid materials, MOFs act as the nucleus or substrate for COF growth. This configuration enhances the stability of the composite structure, which has led to a greater variety of hybrid materials being developed in recent years. Various innovative synthesis approaches have been employed to facilitate COF growth on MOFs, including one‐step reactions, and in situ growth.

The one‐pot strategy involves the simultaneous synthesis of COF components directly on the MOF surface, reducing the complexity associated with multistep procedures. This approach requires careful control of reaction parameters such as solvent choice, temperature, and monomer concentration to ensure the coordinated growth of both MOFs and COFs.

The principle of in situ growth relies on precise regulation of chemical or physical interactions between MOFs and COFs to promote their integration. Tao et al. developed a core–shell heterojunction material with excellent photogenerated carrier separation efficiency and strong visible‐light responsiveness by covalently linking a visible‐light–active COF to an amino‐modified MOF, obtained through the incorporation of 0.225 g of NH_2_‐MIL‐125 during COF synthesis. The resulting composite, TPTi‐0.225, demonstrated remarkable catalytic activity, achieving nearly 99% degradation of propylparaben within 2 h [123].

Another example, preparation of MOF@COF structures via Schiff base chemistry typically begins with the amination of the MOF surface. The aminated MOFs react with aldehyde monomers to form imine (C = N) bonds, creating COF seeds on the MOF surface. Subsequent addition of COF monomers induces the growth of the COF framework on the MOF substrate. Sun et al. synthesized a novel core–shell MOF@COF adsorbent consisting of a Zr‐based MOF core and a COF shell enriched with nitrogen and oxygen functional groups, designed for efficient and selective adsorption of Pd(II). First, amino‐functionalized NH_2_‐UiO‐66 was prepared, providing reactive amino groups for covalent anchoring and direct growth of the COF shell. Subsequently, taking advantage of the strong coordination ability of phosphonate and triazine groups toward Pd(II), a core–shell structure (NH_2_‐UiO‐66@TAP‐COF (M@C)) rich in active sites was synthesized in situ via a Kabachnik–Fields reaction at room temperature. The adsorption performance and mechanism of Pd(II) removal were systematically investigated using M@C, demonstrating its potential for effective Pd(II) elimination [124].

In addition to covalent bonding, π–π stacking interactions between the aromatic systems of MOFs and COFs can also drive the formation of MOF@COF composites. This approach allows hybrid construction even in the absence of amino or aldehyde functional groups, offering an alternative route for material synthesis.

## 4 Applications of Hybrid Metal–Organic Frameworks in Environmental Analysis

Environmental analysis is used to identify and evaluate both internal and external factors that influence the environment. The monitoring process requires highly selective and sensitive tools to identify and/or quantify pollutants in complex matrices [125, 126, 127, 128, 129]. This can be achieved through analyzing different media like air, water, and soil to detect contaminants such as volatile organic compounds, pesticides and heavy metals that can impact health and the environment. In the last decade, many tools were used for accurate and sensitive detection of pollutants such as nanoparticles [130, 131, 132], MIPs [133, 134, 135], MOFs [136, 137, 138, 139], and NZs [140, 141, 142].

Recently, hybrid MOFs have been developed as promising materials for environmental monitoring offering a high degree of robustness, and selectivity. Moreover, these hybrids have the advantages of improving the stability and cost‐effectiveness. The hybrids have many environmental applications such as sensing, photocatalysis or adsorption of contaminants. These smart materials can be applied for complex analyses overcoming the limitations of MOFs alone such as instability in water and poor conductivity [143, 144, 145].

### 4.1 Sensing and Monitoring

Hybrids of MOF with conductive materials like carbon enable electrochemical sensation and monitoring of heavy metals and gases with low detection limits. The MOF binds specific contaminants while the hybrid partner translates this binding into a signal. The hybrids have the advantages of high sensitivity and real‐time detection. Table 3 summarizes the recent sensation applications of hybrid MOFs.

**TABLE 3:** Summary of sensing applications of hybrid MOFs in environmental analysis.

Application	Hybrid type	Outcomes and advantages	Ref.
Metal ions in water	MOF‐Plant Nanobiohybrids	Excellent selectivity and sensitivity (0.05–0.5 μM) for Ag^+^ and Cd^2+^ while the linearity range for Fe^3+^ and Cu^2+^ was 0.05–10 μM	[144]
Trace detection of organophosphorus pesticides in apple juice	Aminated Fe‐based MOF	LOD = 4.55 × 10^−12^ M Excellent sensitivity with good storage stability of one month	[145]
Food nitrite detection	Incorporating magnetic Fe_3_O_4_NPs and AuNPs into the Cu‐MOF	‐ LOD = 0.532 μM ‐ Good selectivity to detect nitrite in ham sausage, squash, milk and brined quail eggs.	[146]
Analysis of phosphates and monitoring of their enzymatic hydrolysis	MOF wrapped Cu nanoclusters‐based fluorescent sensor array	‐ The sensor array generates differentiated fluorescence responses for seven phosphates, including adenosine triphosphate (ATP), cytidine triphosphate, uridine triphosphate, adenosine diphosphate, adenosine monophosphate, pyrophosphate (PPi), and inorganic phosphate. ‐ Enable dynamic monitoring of ATP and PPi hydrolysis.	[147]
Analysis of carbendazim residues in tomato and apple juices	Super‐P carbon (SPCN) black@zeolitic‐imidazolate‐framework‐8 nanocomposite	‐ LOD = 7.79 nM ‐ The fabricated sensor showed good repeatability, reproducibility and specificity	[148]
Vanillin detection in food samples	MOFs and aptamer‐coupling gold nanoparticles	‐ LOD = 3 nM ‐ High sensitivity and selectivity	[149]
Detection of organophosphorus pesticides in food samples	Enzyme‐regulated multifunctional MOFs	‐ LOD =18.7 nM ‐ Rapid and sensitive detection	[150]
Detection of Pb (II) and Cu (II) in different samples	NH_2_‐MIL‐53(Al)/polypyrrole nanocomposite	‐ Excellent sensing performance to Pb (II) and Cu (II) in the range of 1–400 μg/L. ‐ LOD = 0.315 μg/ L and 0.244 μg/L for Pb (II) and Cu (II), respectively.	[151]
Detection of Cd^2+^ and Pb^2+^ in natural water samples	MOF hybrid with multiwalled carbon nanotubes	Linear range of 0.12–2.5 μM for both ions, with LODs of 4.5 nM (Cd^2+^) and 4.9 nM (Pb^2+^).	[152]

Nanomaterials—especially those based on MOFs—show remarkable potential for the development of surface‐enhanced Raman spectroscopy sensors [145]. Their porous structures, simple synthesis processes, abundance of metal active sites and high thermal stability make them highly adaptable and effective materials. They can be used for analyzing multicomponent mixtures in a timely manner. However, the fabrication cost can be challenging!

Zeolite–imidazolate frameworks (ZIFs) exhibit excellent thermal and chemical stability. Among them, ZIF‐8 nanoparticles have been extensively utilized in heavy metal detection and medical applications [148]. Electrochemical techniques are simple and portable; however, their main limitations include low sensitivity, poor stability, and interference from multiple metals. MOF‐based sensors exhibit rapid response times because the efficient electron transfer characteristics enable real‐time detection of Heavy Metal Ions [151].

### 4.2 Photocatalysis

Photocatalysis is a reaction under light irradiation at suitable wavelengths using suitable catalysts to accelerate the rate of chemical reaction [153]. Although MOFs offer notable advantages as photocatalysts, they also face limitations such as large band gaps and the rapid recombination of photogenerated electron–hole pairs. As a result, developing composite catalysts has become a major focus of current research. To address these challenges, researchers have explored coupling MOFs with semiconductors, photosensitizers, or COFs to enhance photocatalytic performance [154].

Photocatalytic water purification is an effective environmental protection technique for removing toxic and harmful substances from industrial wastewater. NH2‐MIL‐101(Fe) was used as an efficient catalyst in the degradation of rhodamine B. The hybrid has an excellent stability due to sustainable release of iron ions [154]. Pharmaceuticals in wastewater represent toxic pollutants and there is a need to monitor their concentrations and effects. Pattappan et al. developed hybrids of MOFs for environmental remediation [155]while Chen et al. proposed a method for antibiotic degradation engineering [156]. The nanocomposite of zeolitic imidazolate frameworks and molybdenum diselenide was used for the degradation of the antibiotics and textile waste from water [157]. Gan et al. discussed the hybrids used for mitigation of pharmaceutical pollutants from aqueous solution [158].

A range of strategies has been applied to MOF composites to strengthen visible‐light absorption, promote more efficient charge‐carrier generation, separation, and transport, and maintain robust recyclability. Table 4 summarizes the recent photocatalysis applications of hybrid MOFs.

**TABLE 4:** Summary of photocatalysis applications of hybrid MOFs in environmental analysis.

Application	Hybrid type	Outcomes and advantages	Ref.
Hydrogen production	ZnCr‐LDH nanosheets	The hybrid has the advantage of improving the photocatalytic activity of hydrogen production under visible‐light with an excellent photo‐chemical stability.	[153]
Water treatment	NH_2_‐MIL‐101(Fe)	‐ Excellent adsorption and Fenton‐like catalytic applications on organic pollutant removal. ‐ 83 % TOC removal in 30 min	[154]
Degradation of acetaminophen and reduction of Cr^6+^	Graphitic carbon nitride/NH_2_‐MIL‐101(Fe) composite	‐ The photodegradation and reduction processes followed first‐order kinetics. ‐ The composite exhibited maximum chromium reduction efficiency of 91% at pH 2.	[155]
Photocatalytic degradation of ciprofloxacin and tetracycline	MoS2/ZIF‐8 hybrid	‐ The good heterojunction in the hybrid results in an efficient charge transfer. ‐ The photocatalyst has many catalytic centers leading to high stability.	[156]
Remediation of textile dye and antibiotic‐contaminated wastewater	Zeolitic imidazolate frameworks with molybdenum diselenide	‐ Degradation of tetracycline hydrochloride and metronidazole showed excellent removal efficiency. ‐ The byproducts of the tetracycline treatment were found to be harmless for aquatic and human life.	[157]
Environmental clean‐up and remediation of pharmaceuticals	COFs with nanomaterials	Removal of diclofenac residues.	[158]
Degradation of methyl orange	TiO_2_ composites with UiO‐66	‐ The degradation of methyl orange was 97.59% at pH = 2 ‐ The enhanced photoactivity is due to the enhanced electron–hole separation.	[159]
Organic pollutant degradation	NH_2_‐MOF‐5/MCOF	‐ Photocatalytic degradation of methylene blue dye with high removal rates. ‐ Excellent reusability and chemical stability over six cycles.	[160]
Degradation of azo dye	bi‐ligand nickle based MOF	‐ Removal of toxic azo dyes from wastewater was achieved using stable and reusable catalyst in 90 seconds. ‐ The reaction kinetics follows pseudo‐first order.	[161]

### 4.3 Adsorption

The MOF hybrids demonstrate enhanced adsorption capacity (Qe) relative to their precursor, which is attributed to the increased presence of biomass‐derived organic functional groups [162, 163]. MOF/cellulose hybrids (CelloMOFs) have significant mechanical characters as tunable porosities and specific surface area making. Cellulose has the advantages of being inexpensive and nontoxic. Moreover, cellulose is insoluble in water, making it ideal to be used in water treatment [164].

Hydrogels were found to be excellent tool for carrying ferricyanate. Their polymer networks are rich in hydrophilic groups such as –OH and –COOH. Thus, hydrogels absorb large amounts of water without dissolving, enabling them to swell and form a flexible gel whose behavior lies between that of a solid and a liquid. Ying et al. developed a novel MOF hydrogel to selectively remove uranyl ions from nuclear wastewater. Infrared spectroscopy and X‐ray photoelectron spectroscopy were used to explain the mechanism of uranium removal [165].

Adsorption of different gases by MOF hybrids has been of interest to many researchers in the last decade. The adsorption has the advantage of being energy‐efficient with minimal operational costs [166]. MOFs had greater application in gas adsorption due to their low hydrostability. Atmospheric carbon dioxide levels increased in the last few years, without effective mitigation strategies, are projected to double by the end of the century, exacerbating environmental degradation and accelerating climate change [167]. Zeeshan et al. synthesized new MOF/Ionic liquids composite which demonstrated 5.7‐ and 45‐fold of CO_2_ uptake and CO_2_/CH_4_ selectivity, respectively, compared to the precursor MOF [168]. Erwin and his team synthesized a hybrid of Ni‐MOF‐5 and activated carbon using microwave with reflux. The gas adsorption capacity showed a linear correlation between the CO_2_ percentage and the amount of CO_2_ captured by the hybrid material. The results also indicated that, when the CO_2_ percentage is held constant, increasing the operating temperature leads to a linear decrease in the amount of CO_2_ adsorbed [169].

Lignin‐based MOFs had many environmental remediation, and energy storage applications. Lignin as a natural polymer is used with metal ions/clusters to form porous structures similar to traditional MOFs. Because lignin is renewable and rich in functional groups, it acts as a ligand, or carbon precursor in MOF synthesis. Lignin can be incorporated into the MOF structure by covalent bonding through functional groups such as phenolic hydroxyl (–OH) or aliphatic hydroxyl (–OH) or carboxyl (–COOH) resulting in high stability. Another mechanism is physical loading through in which MOF crystals grow on lignin surfaces, but without covalent attachment. This simple synthesis has the advantages of low cost and easy to scale. Their exceptional versatility and adaptability make them strong candidates for pollutant removal and renewable energy uses [170]. These materials have also chemical functionality and sustainability with good electrical conductivity after carbonization. They have applied for adsorption of heavy metals and removal of pollutants such as lead, cadmium and chromium. Huiying et al. developed a novel bio‐COF hybrid with lignin from lignocellulose for chromium Cr (VI) remediation. The adsorption behaviors of the smart molecules toward hexavalent chromium were examined under dark conditions and across various ion concentrations. At low initial ion concentrations, the available functional groups can effectively bind with the ions, leading to higher removal efficiency. At higher concentrations, however, the ions exceed the number of active sites, causing the removal efficiency to decline [171]. Table 5 summarizes the recent adsorption applications of hybrid MOFs.

**TABLE 5:** Summary of adsorption applications of hybrid MOFs in environmental analysis.

Application	Hybrid type	Outcomes and advantages	Ref.
Removal of tetracycline from water	ZIF‐67 immobilized on wood aerogel	‐ The reaction follows pseudo‐second‐order kinetics. ‐ The composite demonstrated high tetracycline adsorption capacity of 273.84 mg/g.	[163]
Adsorption removal of Cr(IV) from water	Cellulose aerogels/zeolitic imidazolate framework (ZIF‐8)	‐ Hybrid cellulose aerogels efficiently adsorb heavy metal ions on the surface and bottom from water, with good recyclability. ‐ Langmuir maximum adsorption capacity extends to 41.84 mg/g.	[164]
Removal of uranium from nuclear wastewater	MOF hydrogel with ferrocyanides and functional groups (Fe (∣∣)‐CN‐Fe(∣∣∣), OH, ‐COOH, and ‐NH_2_)	‐ High removal rate with excellent resistance to interference from other ions in complex wastewater samples.	[165]
Separation of carbon dioxide and methane	Incorporating ionic liquids (ILs) into MOFs	‐ The composite is stable even after exposure to water and organic solvents. ‐ The increase in carbon dioxide separation was accompanied by a decrease in methane offering an excellent technique for the separation of the cited gases.	[168]
Adsorption of carbon dioxide	A composite of MOF and activated carbon	The adsorption capacity improved by adding amino groups to the porous surface of the hybrid.	[169]
Adsorption‐photoreduction of chromium ion	A hybrid between COF and lignin	‐ Almost complete Cr(VI) removal was achieved within 2 h. ‐ The hybrid had a maximum adsorption capacity at the range of 5–75 mg/L of Cr(VI).	[171]

Two recent remarkable examples of MOF applications highlight their versatility in gas separation and carbon capture.Wang et al. developed an **isoreticular triazole–MOF series** for one‐step ethylene separation. Conventional MOFs often fail to exhibit the ideal adsorption sequence of CO_2_ > C_2_H_2_ > C_2_H_4_ required for the stepwise removal of impurities. To overcome this limitation, the authors precisely tuned the pore environment within a series of zinc–triazole MOFs. By adjusting the number and position of –NH_2_ and –CH_3_ functional groups on the ligands, the pore chemistry was systematically controlled, transforming the structure from 1D channels to 2D interconnected channels and enabling the desired adsorption selectivity [172].

Similarly, Che et al. reported **hierarchically structured MOF aerogels with tandem pore architectures** for high‐performance CO_2_ capture and separation. These MOF aerogels significantly improved CO_2_ adsorption capacity due to the synergistic effect of multiple pore types. Moreover, the composite aerogel morphology enhances both mechanical stability and mass transfer properties. This improvement is mainly attributed to the cross‐linked aerogel skeleton, which effectively disperses mechanical stress and allows the material to withstand high‐impact conditions [173].

## 5 Role of Artificial Intelligence in Hybrid Modification Strategies of MOFs

Artificial intelligence (AI) holds significant potential to revolutionize the design and optimization of hybrid MOF modification strategies. In the context of MOF hybridization, AI can facilitate the rapid identification of compatible material combinations by leveraging extensive databases of MOF structures and properties. By analyzing pore size distribution, surface area, and topology, AI models can predict which materials such as polymers, nanoparticles, or other inorganic components will work and synergistically enhance MOF performance.

Furthermore, AI‐driven approaches can evaluate the structural stability and interfacial compatibility of hybrid materials by assessing parameters like hydrophobicity, mechanical robustness, and chemical stability. This predictive capability is essential for designing durable composites suitable for industrial applications. AI models can also identify functional groups that optimize specific properties; for instance, suggesting the incorporation of thiol or carboxyl groups groups for heavy‐metal adsorption.

In addition, AI can forecast the pollutant removal efficiency of various MOF hybrids, including organic dyes, pharmaceuticals, and other organic contaminants. By screening thousands of potential hybrid combinations computationally, AI significantly accelerates the discovery process, reducing reliance on time‐consuming and costly experimental trials. Machine learning algorithms, in particular, can analyze complex datasets to uncover hidden correlations and patterns, thereby streamlining the development of high‐performance MOF‐based materials [174, 175, 176].

## 6 Conclusion and Future Perspectives

Postsynthetic modification (PSM) is among the most adaptable methods for incorporating new functionalities into MOFs. Hybrids of MOFs with other materials resulted in enhanced performance of the new smart molecules by integrating the strengths of MOF components with those of other constituents. This review thoroughly discussed the synthesis of MOFs hybrids with carbon dots, NZs, DESs, molecular imprinted polymers, and COFs. The hybrids have many applications like gas storage and separation, drug delivery and sustainable energy. A special focus on their environmental applications was presented through the main mechanisms; sensing, photocatalysis and adsorption.

It's really challenging to employ environmentally friendly techniques to produce green hybrids of MOFs that offer good recyclability and cost‐effectiveness in accordance with the United Nation's Sustainable Development Goals. This requires the use of green solvents, green organic ligands and ionic liquids combined with supercritical CO_2_ to enhance removal efficiency. In the future, synthesis of novel composites using a hybrid of MOFs with materials derived from biowaste is promising in terms of greenness. One of the major challenges is translating small‐scale synthesis in the laboratories into industrial‐scale production. This is due to the difficulty of maintaining consistent pore structure on large scale besides the high operational costs. This can be solved by using continuous‐flow synthesis reactors which provide continuous production.

To minimize MOF degradation in water, researchers can use high‐stability frameworks such as MIL‐101, which is a chromium‐based MOF composed of chromium (III) ions and terephthalate linkers. It has the advantages of large pore volume and chemical stability, making it a widely studied for adsorption and catalysis applications. Improving the long‐term stability in natural environments can be achieved through polymer encapsulation. This involves polymer coating of MOF particles which improves mechanical durability and prevents hydrolysis. The stability of MOFs hybrids is dependent on metal–ligand bond strength. Researchers can improve this by using metal ion exchange and doping with stronger metals.

AI‐driven strategies could be a promising tool for the next generation of MOF revolution. High‐throughput computational screening methods based on molecular simulations would play a key role in producing a large number of composites. The most important limitations are the cost and the requirement for specialized expertise. Moreover, many databases exhibit bias because they rely on a limited set of building blocks to construct hypothetical structures. The MOF community needs to eliminate barriers to data access and utilization by offering user‐friendly databases, training resources, and relevant data analytics platforms.

## Author Contributions

**Samar H. Elagamy**: data curation, conceptualization, methodology, supervision, writing – original draft. **Adel M. Michael**: data curation, conceptualization, writing – original draft. **Reem H. Obaydo**: conceptualization, visualization, writing – review and editing. **Hayam M. Lotfy**: supervision, conceptualization, writing – original draft, writing – review and editing.

## Declaration of AI Assistance

In preparing this manuscript, we used AI‐based tools for language refinement and grammar checking to improve readability and clarity. Additionally, AI‐assisted software was employed to enhance the resolution of figures for better visualization. We confirm that the use of these tools did not influence the scientific content, data interpretation, or conclusions of this work. All intellectual contributions, analyses, and conclusions presented in this manuscript are solely those of the authors.

## Funding

The authors have nothing to report.

## Conflicts of Interest

The authors declare no conflicts of interest.

## Data Availability

All data generated or analyzed during this study are included in this article.
